# Epidemics of Drug-Resistant Bacterial Infections Observed in Infectious Disease Surveillance in Japan, 2001-2005

**DOI:** 10.2188/jea.17.S42

**Published:** 2008-01-30

**Authors:** Michiko Izumida, Masaki Nagai, Akiko Ohta, Shuji Hashimoto, Miyuki Kawado, Yoshitaka Murakami, Yuki Tada, Mika Shigematsu, Yoshinori Yasui, Kiyosu Taniguchi

**Affiliations:** 1 Department of Public Health, Saitama Medical University Faculty of Medicine.; 2 Department of Hygiene, Fujita Health University School of Medicine.; 3 Department of Health Science, Shiga University of Medical Science.; 4 Infectious Disease Surveillance Center, National Institute of Infectious Diseases.

**Keywords:** Drug-resistant Bacterial Infections, Methicillin-resistant *Staphylococcus aureus* (MRSA), Multi-drug-resistant *Pseudomonas Aeruginosa* (MDRPA), Penicillin-resistant *Streptococcus Pneumoniae* (PRSP), Sentinel Surveillance

## Abstract

**BACKGROUND:**

Drug-resistant bacteria have been increasing together with advancement of antimicrobial chemotherapy in recent years. In Japan, the target diseases in the National Epidemiological Surveillance of Infectious Diseases (NESID) include some drug-resistant bacterial infections.

**METHODS:**

We used the data in the NESID in Japan, 2001-2005. Target diseases were methicillin-resistant *Staphylococcus aureus* (MRSA), penicillin-resistant *Streptococcus pneumoniae* (PRSP) and multi-drug-resistant *Pseudomonas aeruginosa* (MDRPA) infections. The numbers of patients reported by sentinel hospitals (about 500) on a monthly basis were observed.

**RESULTS:**

The numbers of patients per month per sentinel hospital of 2001-2005 were 3.37-3.98 in MRSA, 0.96-1.19 in PRSP, and 0.11-0.13 in MDRPA infections. The sex ratios (male / female) of patients were 1.69-1.82, 1.34-1.43, and 1.71-2.52, respectively. More than 50% of all patients were adults aged 70 years or older in MRSA and MDRPA infections, but more than 60% were children under 10 years in PRSP infections. The number of patients per sentinel hospital in MRSA infections showed little variation between months, but evidenced a large variation in PRSP and MDRPA infections. The annual trend in the number of patients per sentinel hospital was increasing significantly for the 5-year period in MRSA and PRSP infections, but not in MDRPA infections.

**CONCLUSIONS:**

We revealed sex-age distributions of the patients reported to NESID in Japan, 2001-2005. An increasing incidence of MRSA and PRSP infections and monthly variation in PRSP and MDRPA infections were observed for the 5-year period. Extended observation would be necessary to confirm these trends and variations.

Drug-resistant bacteria have been increasing together with advancement of antimicrobial chemotherapy in recent years.^1^ The emergence of drug-resistant bacteria will make these infections more difficult to treat.^1^^-^^5^ Observing the epidemics of drug-resistant bacterial infections is necessary and important.^1^ In Japan, the target diseases in the National Epidemiological Surveillance of Infectious Diseases (NESID) include methicillin-resistant *Staphylococcus aureus* (MRSA) infections, penicillin-resistant *Streptococcus pneumoniae* (PRSP) infections, and multi-drug-resistant *Pseudomonas aeruginosa* (MDRPA) infections.^6^ According to a current report in Japan, 57.1% (70.9% of inpa-tients, 31.2% of outpatients) of *S. aureus* isolated are resistant to methicillin, and 62.4% (61.5% of inpatients, 63.8% of outpatients) of *S. pneumoniae* isolated are resistant to penicillin.^7^ In *P. aeruginosa*, about 80% of these isolated bacteria are susceptible to imipenem or ciprofloxacin, and 90% are susceptible to amikacin.^7^ There are few reports about the epidemiologic features and changes of incidence in these infections nationwide in Japan.

In the present study, we observed the number of patients reported to NESID in Japan, 2001-2005, and revealed sex-age distributions of the patients and temporal changes in the number of patients of these three drug-resistant bacterial infections.

## METHODS

### Surveillance of Infectious Diseases in Japan

The NESID in Japan has been described elsewhere.^6^^,^^8^^-^^9^ The number of drug-resistant bacterial infections at sentinel hospitals is reported every month to public health centers.^6^^,^^8^^-^^9^ The sentinel hospitals (about 500 hospitals with more than 300 beds providing medical care in pediatrics and internal medicine across Japan) primarily target inpatients.^6^^,^^8^ The information reported includes sex and age.^6^

Reporting criteria of bacteriological examinations of these infections were *S. aureus* resistant to oxacillin [minimal inhibitory concentration (MIC)≧4*µ*g/mL] for MRSA, *S. pneumoniae* resistant to penicillin [MIC≧0.125*µ*g/mL] for PRSP, and *P. aeruginosa* resistant to imipenem [MIC≧16*µ*g/mL], amikacin [MIC≧32*µ*g/mL] and ciprofloxacin [MIC≧4*µ*g/mL] for MDRPA.^6^

### Surveillance Data and Method of Analysis

We used the data in the NESID in Japan, 2001-2005. Target diseases are three infections, MRSA, PRSP and MDRPA.

Annual trend and monthly variation in the number of patients per sentinel hospital were evaluated using a Poisson regression with that as a dependent variable, and a year (as a continuous variable) and a month (as dummy variables) as independent variables. The SAS^®^ (SAS Institute, Cary, North Carolina, USA) GENMOD procedure was used for the analysis.

## RESULTS

Table 1 shows the numbers of patients with drug-resistant bacterial infections reported by sentinel hospitals in 2001-2005. The total numbers of patients (per month per sentinel hospital) were 18,257-22,454 (3.37-3.98) in MRSA infections, 5,202-6,700 (0.96-1.19) in PRSP infections, and 608-747 (0.11-0.13) in MDRPA infections. The sex ratios (male / female) of patients were 1.69-1.82, 1.34-1.43, and 1.71-2.52, respectively.

**Table 1.** Numbers of patients with drug-resistant bacterial infections reported by sentinel hospitals in 2001-2005.

Diseases	patients	Year				

		2001	2002	2003	2004	2005
MRSA infections	Total	18,257	19,904	21,117	21,835	22,454
	Male	11,482	12,638	13,637	13,828	14,215
	Female	6,775	7,266	7,480	8,007	8,239
	Male/Female	1.69	1.74	1.82	1.73	1.73
	Number of patients per month per sentinel hospital	3.37	3.59	3.77	3.87	3.98
					
PRSP infections	Total	5,202	6,071	6,400	6,700	6,217
	Male	3,043	3,497	3,660	3,893	3,660
	Female	2,159	2,574	2,740	2,807	2,557
	Male/Female	1.41	1.36	1.34	1.39	1.43
	Number of patients per month per sentinel hospital	0.96	1.09	1.14	1.19	1.10
					
MDRPA infections	Total	608	715	747	669	692
	Male	398	496	535	422	452
	Female	210	219	212	247	240
	Male/Female	1.90	2.26	2.52	1.71	1.88
	Number of patients per month per sentinel hospital	0.11	0.13	0.13	0.12	0.12

MRSA: methicillin-resistant *Staphylococcus aureus*.

PRSP: penicillin-resistant *Streptococcus pneumoniae*.

MDRPA: multi-drug-resistant *Pseudomonas aeruginosa*.

**Figure 1.**
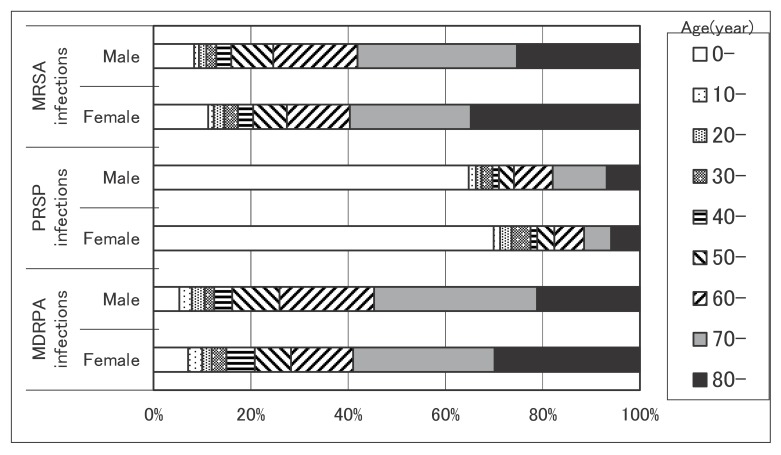
Age distributions of drug-resistant bacterial infections by sex. MRSA: methicillin-resistant *Staphylococcus aureus*. PRSP: penicillin-resistant *Streptococcus pneumoniae*. MDRPA: multi-drug-resistant *Pseudomonas aeruginosa*.

Figure 1 shows the age distributions of drug-resistant bacterial infections by sex. More than 50% of all patients were adults aged 70 years or older in MRSA and MDRPA infections, but more than 60% of them were children under 10 years in PRSP infections.

**Figure 2.**
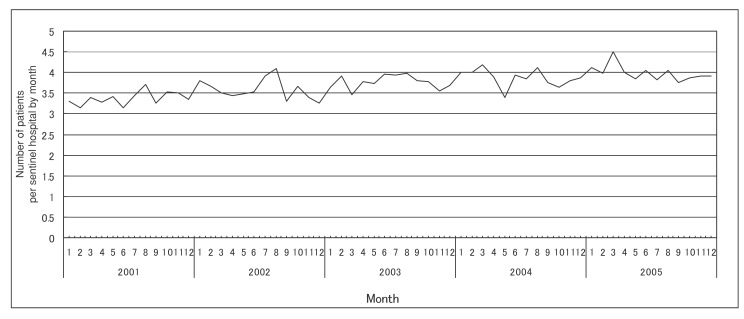
Number of patients per sentinel hospital of methicillin-resistant *Staphylococcus aureus* infections by month (2001-2005).

**Figure 3.**
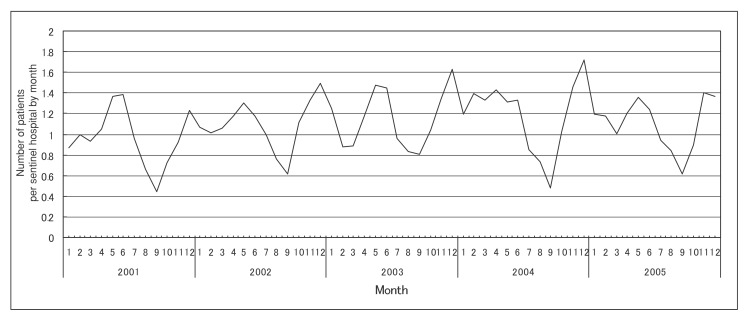
Number of patients per sentinel hospital of penicillin-resistant *Streptococcus pneumoniae* infections by month (2001-2005).

**Figure 4.**
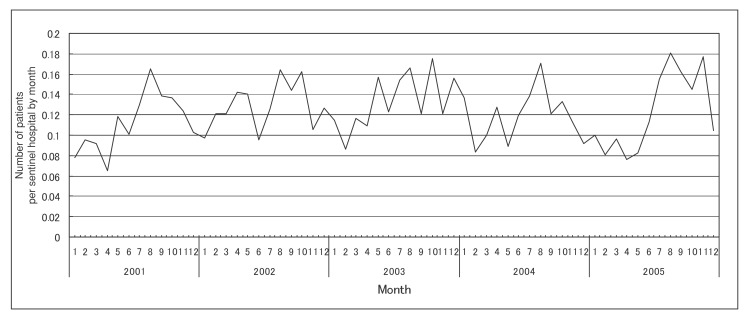
Number of patients per sentinel hospital of multi-drug-resistant *Pseudomonas aeruginosa* infections by month (2001-2005).

Figures 2, 3, and 4 show the number of patients per sentinel hospital of MRSA, PRSP and MDRPA infections by month, respectively. Table 2 shows the adjusted ratios of the number of patients per sentinel hospital by year and month in Japan, 2001-2005. The number of patients per sentinel hospital of MRSA infections showed little variation between months (adjusted ratio: 0.96-1.07 compared with the annual mean value), but the annual trend in the number of patinets per sentinel hospital was increasing significantly (adjusted ratio: 1.04 for 1 year, that is equal to 1.23 for 5 years) (Figure 2 and Table 2). The number of those PRSP infections showed a large variation between months (adjusted ratio: 0.55-1.40 compared with the annual mean value), and their annual trend was increasing significantly (adjusted ratio: 1.03 for 1 year, that is equal to 1.19 for 5 years). The month with the least number of patients was September, and the month with the largest number was December, followed by May (Figure 3 and Table 2). In MDRPA infections, the number of patients per sentinel hospital showed a large variation between months (adjusted ratio: 0.77-1.40 compared with the annual mean value), and it was higher during the latter than the former half of the year. However, their annual trend was not increasing significantly (adjusted ratio: 1.01 for 1 year, that is equal to 1.05 for 5 years) (Figure 4 and Table 2).

**Table 2.** Adjusted ratios of the number of patients per sentinel hospital by year and month in 2001-2005.

Parameter	MRSA infections		PRSP infections		MDRPA infections	
		
	Adjusted ratio^*^ of number of patients per sentinel hospital	p-value	Adjusted ratio^*^ of number of patients per sentinel hospital	p-value	Adjusted ratio^*^ of number of patients per sentinel hospital	p-value
Year	1.04	< 0.001	1.03	< 0.001	1.01	0.483
						
January	1.02	0.124	1.05	0.017	0.87	0.022
February	1.01	0.463	1.02	0.237	0.77	< 0.001
March	1.03	0.010	0.98	0.266	0.87	0.018
April	0.99	0.298	1.13	< 0.001	0.86	0.014
May	0.96	< 0.001	1.28	< 0.001	0.97	0.546
June	1.00	0.661	1.23	< 0.001	0.91	0.116
July	1.02	0.041	0.88	< 0.001	1.16	0.006
August	1.07	< 0.001	0.72	< 0.001	1.40	< 0.001
September	0.96	< 0.001	0.55	< 0.001	1.13	0.022
October	0.99	0.498	0.90	< 0.001	1.24	< 0.001
November	0.98	0.028	1.21	< 0.001	1.05	0.346
December	0.97	0.011	1.40	< 0.001	0.96	0.452

MRSA: methicillin-resistant *Staphylococcus aureus*.

PRSP: penicillin-resistant *Streptococcus pneumoniae*.

MDRPA: multi-drug-resistant *Pseudomonas aeruginosa*.

* Adjusted ratio was estimated by Poisson regression analysis.

The ratio for the year indicates the difference in one year. The ratio for month is the ratio to the annual mean value.

## DISCUSSION

We could observe representative cases of three major drug-resistant bacterial infections throughout Japan because we used the data from the sentinel hospitals in the NESID. But the patients in the present study might be more serious than all patients with these infections because these hospitals primarily target inpatients.

Half or more of the MRSA and MDRPA patients in our study were elderly. The two bacteria are basically opportunistic and hospital pathogens.^10^^-^^11^ Besides, the elderly generally visit hospitals more than other adults, and they may easily become compromised hosts. On the other hand, more than 60% of all patients of PRSP infections involved children under 10 years. *S. pneumoniae* colonizes in the nasopharynx of healthy children more than healthy adults, and causes infections of the middle ear, sinuses, trachea, bronchi, and lungs.^12^ Infants may have an increased risk of viral (upper respiratory tract) infections (which triggered *S. pneumoniae* infections) compared to adults. We consider these to be why most patients of PRSP infections are in children.

In Japan, although there are many reports about annual changes in the susceptibilities of bacteria isolated from patients,^1^^,^^13^^-^^18^ only a few concern annual changes in the incidence of infections due to drug-resistant bacteria, and even fewer with monthly variations in incidence.

In the present study, the annual trend in the number of patients per sentinel hospital was found to be increasing significantly in MRSA and PRSP infections, but not in MDRPA infection for the 5-year period between 2001 and 2005. A past report showed that a proportion of PRSP in *S. pneumoniae* which was isolated from patients (with lower respiratory infectious diseases) was increasing in recent years.^15^ The increasing trend of PRSP infections might reflect an increasing proportion of PRSP in *S. pneumoniae.* However, one should recall that the number of reported patients in sentinel hospitals might increase if the number of population covered by the sentinel hospitals increased. In addition, if the clinical abilities of pediatrics departments in the sentinel hospitals were improved, the number of patients might increase because many patients with PRSP infections are children. On the other hand, the above-mentioned report showed that a proportion of MRSA in *S. aureus* which was isolated from patients was not increasing in recent years.^15^ We thought one of the reasons for the increasing trend of MRSA infections was that the number of compromised hosts who would easily become MRSA-infected was increasing for the observed period, but we did not know the details. In MDRPA infections, because the number of patients per sentinel hospital was very few, it is important to observe the longer period trend.

In addition, in the present study, the numbers of patients per sentinel hospital showed a large monthly variation in PRSP and MDRPA infections, possibly reflecting differences in their monthly incidence. PRSP is frequently isolated from children who have acute otitis media. There was a report that the number of children who were receiving treatment in a hospital as inpatients for acute otitis media peaked in December and May, and was lowest in September.^19^ The monthly variation of PRSP infections in our study agrees with this variation. On the other hand, we could not find previous reports that the incidence of MRSA infections showed a large monthly variation, and our result was consistent with this. There was no report that the incidence of MDRPA infections showed a large monthly variation, and the reasons for the large monthly variation in our study remain unknown. Because the number of patients was very few in MDRPA infections, the longer period data might be effective to observe a clear monthly variation in incidence.

In conclusion, in the present study we revealed the sex-age distributions of the patients reported to NESID in Japan, 2001-2005. An increasing incidence of MRSA and PRSP infections and monthly variation in PRSP and MDRPA infections were observed for the 5-year period. Extended observation would be necessary to confirm these trends and variations.
